# Prevalence and clinical profile of COVID-19 among farmworkers from the interior of the state of Rio Grande do Sul, Brazil

**DOI:** 10.47626/1679-4435-2025-1444

**Published:** 2025-09-14

**Authors:** Carlos Henrique de Lemos Muller, Hildegard Hedwig Pohl, Miriam Beatrís Reckziegel

**Affiliations:** 1 Interprofessional Residency Program in Healthcare: Urgency and Emergency, Hospital Santa Cruz, Santa Cruz do Sul, RS, Brazil.; 2 Postgraduate Program in Health Promotion, Department of Physical Education and Health, Universidade de Santa Cruz do Sul (UNISC), Santa Cruz do Sul, RS, Brazil.

**Keywords:** COVID-19, post-acute COVID-19 syndrome, rural workers, covid-19, síndrome de covid-19 pós-aguda, trabalhadores rurais

## Abstract

**Introduction:**

Farmworkers in Brazil face poor living conditions, limited healthcare access, and high prevalence of chronic diseases. These vulnerabilities may increase COVID-19 risk, complications, and persistent symptoms, underscoring the importance of characterizing the disease’s impact in this population.

**Objectives:**

To identify the prevalence and clinical profile of COVID-19 among farmworkers of cities that participate of the Conselho Regional de Desenvolvimento do Vale do Rio Pardo.

**Methods:**

A retrospective cross-sectional study that utilized a database from a research performed with rural workers of cities from Conselho Regional de Desenvolvimento do Vale do Rio Pardo. Hundred seven volunteers were included (54.01 ± 13.02 years) who answered the questionnaries of life style and COVID-19, in addition to having performed body composition assessment. Prevalence ratio was measured to verify association between risk factors and COVID-19 diagnosis.

**Results:**

Twenty-five people (23.36%) were diagnosticated with COVID-19. The more described symptoms were fatigue (84%), fever (68%), cough (68%), loss of taste (68%), headache (68%), and sore throat (64%). None of the participants was hospitalized. Symptoms of long covid were observed in 52% participants, with fatigue (24%) and breathless (16%) being the most prevalent. The results showed positive association between COVID-19 diagnosis and hypertension (prevalence ratio 1.23), cancer (prevalence ratio 1.22) and obesity (prevalence ratio 1.76).

**Conclusions:**

The results help to characterize the clinical profile of the disease in a population with less access to health services.

## Abstract (pt)

**Introdução:**

Trabalhadores rurais no Brasil enfrentam condições precárias de vida, acesso limitado à saúde e alta prevalência de doenças crônicas. Essas vulnerabilidades podem aumentar o risco de covid-19, complicações e sintomas persistentes, ressaltando a importância de caracterizar o impacto da doença nessa população.

**Objetivos:**

Identificar a prevalência de covid-19 e seus agravos em trabalhadores rurais de municípios integrantes do Conselho Regional de Desenvolvimento do Vale do Rio Pardo.

**Métodos:**

Estudo transversal retrospectivo que utilizou o banco de dados de pesquisa conduzida com trabalhadores rurais de municípios integrantes do Conselho Regional de Desenvolvimento do Vale do Rio Pardo. Foram incluídos 107 sujeitos (54,01 ± 13,02 anos) que responderam aos questionários de estilo de vida e de covid-19 e realizaram avaliação da composição corporal. A razão de prevalência foi calculada para verificar a associação entre fatores de risco e o diagnóstico de covid-19.

**Resultados:**

No total, 25 indivíduos (23,36%) foram diagnosticados com covid-19. Os sintomas mais relatados foram cansaço (84%), febre (68%), tosse (68%), perda do paladar (68%), dor de cabeça (68%) e dor de garganta (64%). Nenhum dos participantes foi hospitalizado. A covid longa foi verificada em 52% dos sujeitos, com cansaço (24%) e falta de ar (16%) sendo os sintomas mais prevalentes. Os resultados indicam associação positiva entre diagnóstico de covid-19 e hipertensão (razão de prevalência = 1,23), câncer (razão de prevalência = 1,22) e obesidade (razão de prevalência = 1,76).

**Conclusões:**

Os dados coletados no presente estudo ajudam a caracterizar o perfil da doença em uma população com menor acesso a serviços de saúde.

## Introduction

The population of Brazil exceeds 203 million inhabitants,^1^ 19 million of whom are farmworkers.^2^ In many cases, their living conditions include reduced access to education and employment activities involving great physical exertion, long working hours, and working for many days consecutively.^3^ Compounding these issues, scant access to health services also contributes to health problems in these populations.^4^

The unreliable access to health services faced by such populations is an additional complicating factor during situations in which demand for treatment is elevated, as was observed during the COVID-19 pandemic. In this disease, infection by the virus can cause asymptomatic cases but can also provoke development of severe acute respiratory syndrome.^5^ Other complications include activation of a systemic inflammatory process, cardiovascular and thromboembolic events, and even shock.^6^ In many cases, patients need to be admitted to hospital and, in some cases, must be admitted to an intensive care unit and need to be put on mechanical ventilation, which is associated with a high mortality rate.^5^

Since the onset of the pandemic, studies have identified risk factors that can increase the likelihood of infection, complications of the disease, and mortality.^7^-^10^ These factors include age, smoking, obesity, cardiovascular diseases, severe lung disease, type 2 Diabetes mellitus, chronic kidney disease, and clinical conditions that result in immunosuppression.^7^-^10^ In this regard, the literature contains evidence of high prevalence rates of hypertension, dyslipidemia, and multimorbidity among farmworkers,^11^ exceeding rates observed in studies of the general population.^12^ These findings suggest that farmworkers may be at greater risk of complications if they are infected by the virus.

Another factor that has drawn the attention of the scientific community is persistence of symptoms after COVID-19. These residual symptoms may be respiratory (breathlessness and coughing), cardiovascular (chest pains and palpitations), neurological (cognitive impairment, anxiety, and headaches), muscular (fatigue and joint pain), and gastrointestinal (abdominal pains, nausea, and diarrhea).^13^ It is extremely important to identify these symptoms among farmworkers because they can have negative impacts on both their daily lives and their occupational activities.

Considering all of the above, farmworkers may be more exposed to COVID-19 infection and may also be at greater risk of complications, so it is extremely important to characterize the profile of the disease in this population. As such, the objective of this study was to identify the prevalence of COVID-19 and its negative effects (symptoms, hospital admissions, and persistent symptoms) among farmworkers in the municipal districts that are members of the Vale do Rio Pardo Regional Development Council (COREDE-VRP).

## Methods

### Study area and population

This is a retrospective cross-sectional study, with a convenience sample that analyzed the database from a previous study conducted with farmworkers from towns that participate in the COREDE-VRP. The project was approved by the research ethics committee at the Universidade de Santa Cruz do Sul (UNISC), under decision number 7,177,664. The study enrolled farmworkers over the age of 18 years who had completed questionnaires on lifestyle and COVID-19 and underwent a body composition assessment. Participants were excluded from the study if they had not answered one of the questionnaires or if the database did not contain data on their body composition.

### Data collection procedures

Data were extracted from a database compiled for a research project entitled *Triagem de fatores de risco relacionados àobesidade, estilo de vida, saúde cardiometabólica e doenças crônicas não transmissíveis: impacto da promoção e educação em saúde em trabalhadores rurais e urbanos – Fase IV* [Screening for risk factors related to obesity, lifestyle, cardiometabolic health, and non-transmissible chronic diseases: impact of health promotion and education among rural and urban workers– Phase IV], conducted in 2021 and 2022. The following variables were used to profile the sample: age, sex, body mass, fat mass and muscle mass, body fat percentage, and body mass index (BMI), in addition to data on comorbidities and medications.

The main study, for which the database was compiled, was conducted at the UNISC Physical Activity Laboratory. On the assessment day, volunteers underwent anthropometric measurements and completed the lifestyle and COVID-19 questionnaires. The variables of interest exported from the database were obtained from the answers to the COVID-19 questionnaire. The primary outcome was a COVID-19 diagnosis and the secondary outcomes included related symptoms, hospital admissions, and persistence of symptoms after recovery from COVID-19.

### Body composition assessment

Body mass and height were measured using a balance with a built-in stadiometer (Welmy SA, Santa Bárbara do Oeste, Brazil). BMI was then calculated as follows: BMI = body mass/height^2^. Body fat percentage and muscle mass were measured by bioelectrical impedance analysis (BIA) using an InBody 720 device (Biospace, Seoul, South Korea), in accordance with the manufacturer’s instructions.^14^ Volunteers were instructed to adhere to the following recommendations: not to eat before the test; use the toilet before the test; wear light clothing; remove adornments and metallic objects; not to engage in physical activity before the test; not to bathe before the test; stand upright for 5 minutes before the test; and not to ingest diuretic substances before the test.

For the bioimpedance analysis, muscle mass is calculated as follows: [(height^2^ / BIA resistance × 0.401) + (sex × 3.825) + (age × -0.071)] + 5.102, as validated by Janssen et al.,^15^ where BIA resistance is expressed in Ohms; sex is coded as male = 1 and female = 0; and age is expressed in years. The result is the muscle mass percentage.

### Lifestyle questionnaire

Lifestyle data were obtained using a questionnaire^16^ comprising items on identity, lifestyle, physical activity, and health indicators. The following variables were used for the present study: sex, age, comorbidities, medications used, smoking, and physical exercise.

### COVID-19 questionnaire

This questionnaire was used to obtain information on COVID-19 among the study subjects. It asked: a) if the respondent had been diagnosed with COVID-19 and the number of times; b) what symptoms they had had; c) whether they had been admitted to hospital; and d) if any symptoms had remained after recovery.

### Statistical analysis

Data were analyzed using the Statistical Package for the Social Sciences (IBM SPSS v.25.0, Armonk, NY, USA). The normality of data was assessed using the Shapiro-Wilk test. Results for continuous variables were expressed as mean and standard deviation for parametric data and as median and interquartile range for nonparametric data. Categorical variables were reported as frequencies and percentages. Prevalence ratios (PR) were calculated to test for associations between diagnosis of COVID-19 and the following risk factors: obesity, diabetes, cancer, arterial hypertension, and smoking. The PRs were calculated by dividing the prevalence among the exposed group by the prevalence among the group that was not exposed to each risk factor.

## Results

A total of 112 farmworkers answered the lifestyle questionnaire. Five of these were excluded from the analysis because they had not attended the body composition assessment, resulting in a final sample of 107 people. The sample characteristics are shown in Table 1. The mean age of the sample was 54.01 ± 13.02 years, indicating that most of them were not elderly; mean BMI was in the overweight range (28.11 ± 4.64 kg/m^2^); and mean body fat percentage was high (31.23 ± 9.52%). Lifestyle data revealed that the majority of participants did not engage in physical activity and did not smoke. The most common health problems were hypertension (38.31%), dyslipidemia (38.31%), and depression (30.28 %). In line with this, the most common medications taken by the participants were antihypertensives and antilipemics.

**Table 1:** Sample characteristics (n = 107)

Variable	Value (%)
Age (years)	54.01 ± 13.02
Sex	
Male	55 (51.40)
Body composition	
Body mass (kg)	78.08 ± 14.17
Muscle mass (kg)	50.27 ± 9.78
Fat mass (kg)	24.78 ± 10.23
Body fat percentage (%)	31.23 ± 9.52
Body mass index (kg/m^2^)	28.11 ± 4.64
Physical activity?	
Yes	35 (32.71)
No	72 (67.29)
Smoking	
Never smoked	80 (74.76)
Quit	20 (18.69)
Smoker	7 (6.55)
Health problems	
Arterial hypertension	41 (38.31)
Dyslipidemia	41 (38.31)
Depression	30 (28.03)
Diabetes	11 (10.00)
Cancer	11 (10.00)
Kidney disease	10 (9.09)
Lung disease	4 (3.63)
Acute myocardial infarction	2 (1.81)
Heart failure	2 (1.81)
Stroke	1 (0.90)
Medications	
Hydrochlorothiazide	17 (15.45)
Enalapril	12 (11.21)
Losartan	9 (8.41)
Simvastatin	8 (7.27)
Atenolol	6 (5.45)
Levothyroxine	5 (4.54)
Acetylsalicylic acid	4 (3.63)

Continuous variables expressed as mean ± standard deviation and categorical variables expressed as frequency and percentage.

Twenty-five of the study participants had been diagnosed with COVID-19, the majority (72%) of whom had only had one positive diagnosis. The most frequently reported symptoms were tiredness (84%), fever (68%), coughing (68%), loss of sense of taste (68%), headache (68%), and sore throat (64%). None of the participants had needed to be admitted to hospital because of COVID-19. Symptoms that remained after recovery were reported by 52% of participants, with tiredness (24%) and shortness of breath (16%) the most prevalent. Data on COVID-19 in the sample are shown in Table 2. 

**Table 2:** Data on COVID-19 in the sample (n = 107)

Variables	Value (%)
Diagnosed with COVID-19	
Yes	25 (23.36)
Number of positive diagnoses	25 (100.00)
1	18 (72.00)
2	6 (24.00)
3	1 (4.00)
Symptoms	
Tiredness	21 (84.00)
Fever	17 (68.00)
Coughing	17 (68.00)
Loss of taste	17 (68.00)
Headache	17 (68.00)
Sore throat	16 (64.00)
Loss of smell	13 (52.00)
Dyspnea	7 (28.00)
Chest pain	5 (20.00)
Diarrhea	2 (8.00)
Residual symptoms after recovery	
Yes	13 (52.00)
Symptoms that remained	
Tiredness	6 (24.00)
Shortness of breath	4 (16.00)
Loss of memory	2 (8.00)
Sore throat	2 (8.00)
Headache	1 (4.00)
Risk factors	PR
Obesity	1.76
Hypertension	1.23
Cancer	1.22
Diabetes	0.36

Categorical variables expressed as frequency and percentage. PR = prevalence ratio.

Prevalence ratios were calculated to test for associations between risk factors and COVID-19 diagnosis. The following risk factors were considered: obesity, diabetes, arterial hypertension, cancer, and smoking. The results indicate positive associations between COVID-19 and hypertension (PR = 1.23), cancer (PR = 1.22), and obesity (PR = 1.76). In contrast, there was a negative association between diagnosis of COVID-19 and diabetes (PR = 0.36). Although it had been planned to calculate a PR for smoking and COVID-19, this was not possible, since none of the smokers had been diagnosed with the disease.

## Discussion

This study was designed to conduct descriptive analyses to identify the prevalence of COVID-19 and its negative effects among farmworkers from Southern Brazil. The results indicated a prevalence of 23.26% of participants diagnosed with COVID-19 and the most common symptoms were tiredness, fever, coughing, loss of sense of taste, headache, and sore throat. None of those diagnosed with COVID-19 needed to be admitted to hospital. Residual symptoms after recovery were observed in 52% of the sample, the most prevalent of which were tiredness and shortness of breath. Tests for associations between risk factors and diagnosis revealed positive associations with hypertension, cancer, and obesity.

Comparing the prevalence of COVID-19 in the study sample with the rate in the Brazilian population revealed similar values. Using data published in 2024 by the Brazilian Institute of Geography and Statistics (IBGE - *Instituto Brasileiro de Geografia e Estatística*) stating the Brazilian population at 203 million inhabitants and data from the Coronavirus Panel on the number of cases in the country, (38,991,809), the overall prevalence rate was calculated at 19.20%, while the rate in our study was 23.26%. Studies investigating the prevalence among farmworkers in the United States reported rates of 12.70%^17^ and 9.30%,^18^ both lower than in our study. The study by Lewnard et al.^17^ observed a significantly higher proportion of individuals (41.20%) who reported symptoms associated with the disease, but without a confirmed diagnosis. This could suggest that the number of people infected by the virus was greater than that estimated by the study.^17^ Notwithstanding, there is also the possibility that people considered that symptoms characteristic of the disease (headache, shortness of breath, loss of sense of taste and smell, among others) were caused by other factors and thus did not get diagnosed.^19^ These factors may explain the differences between the cited studies.

In this study, six symptoms (tiredness, fever, coughing, loss of sense of taste, headache, and sore throat) were most prevalent among people diagnosed with COVID-19. In a study by Abebe et al.,^20^ fever, coughing, and tiredness were the most common symptoms among symptomatic individuals with mild and moderate cases of the disease. In a study conducted with farmworkers in the US, fever and loss of sense of taste were also identified as among the main symptoms, in common with the present study.^17^ In the Epicovid-19 national Brazilian study, fever, coughing, and headache were also among the most often reported symptoms, both among a group of people with non-transmissible chronic diseases and among those without these conditions.^21^ Additionally, loss of sense of smell and muscle pains were also identified as symptoms in the Epicovid-19 study, which was not the case in our study.

None of the individuals in the present study who had COVID-19 needed to be admitted to hospital. Hospitalization is associated with severe forms of the disease, manifesting with pneumonia with hypoxemia (SpO2 < 92%).^20^ Additionally, studies have shown that certain comorbidities (hypertension, cancer, obesity, diabetes, and pulmonary diseases) are related to the severity of the disease and, as a consequence, to hospital admission.^22^-^24^ While no cases of hospital admission were observed in the present study, according to the PRs calculated, some comorbidities (hypertension, cancer, and obesity) were associated with infection by COVID-19. Thus, those individuals in the sample who had these risk factors appear to have been more susceptible to infection by the virus. The strongest association calculated was with obesity (PR = 1.76). This finding confirms other studies that also identified obesity as a factor predisposing to infection, with risk increasing as BMI and visceral adipose tissue increase.^8^,^22^ A study conducted in Jordan with 2,148 participants found that age, having three or more comorbidities, and obesity were predictors of disease severity and hospital admission.^23^ A systematic review and meta-analysis also identified increased rates of infection and hospital admissions among obese patients.^22^ Even having found this association, one possible reason for the absence of hospital admissions among the participants in this sample is that these individuals’ occupational activities require great physical effort, which keeps them physically active even without engaging in structured physical exercise. A possible consequence of this is a reduction in the incidence of COVID-19 infection compared with completely sedentary people.^25^

In this study, residual symptoms post-COVID-19 were observed in 52% participants, in contrast with other studies.^26^-^28^ A study that investigated long covid 6 months after hospital admission found that 76.0% of subjects exhibited symptoms^27^ and another found symptoms in 13.3% of people 28 days after recovery.^28^ The overall prevalence of post-covid syndrome was 43.0% of the population and was higher among people who had been hospitalized than in those who did not need hospital admission (51.0% *vs.* 34.0%).^26^ These differences could be because of the time after the disease at which symptoms were assessed and by the degree of severity. The symptoms of post-covid syndrome that were most prevalent in the present study (tiredness and shortness of breath) were also observed in other studies.^26^-^28^ In contrast to our study, other studies observed headache, loss of sense of smell,^28^ joint pains,^26^ problems sleeping,^26^,^27^ and problems with memory.^26^

The importance of the present study lies in the fact that, to the best of our knowledge, it is the first to characterize the profile of COVID-19 among farmworkers, who constitute a population with a dearth of studies focused on their health. Moreover, calculating PRs enabled a more in-depth analysis of the risk factors that could have increased susceptibility to infection by the virus. Nevertheless, this study does have some limitations. First, self-report COVID-19 data were used, which are dependent on subjects’ memory and could introduce memory bias. Second, the questions on COVID-19 were administered at different times after recovery, which could have affected reporting of long covid symptoms. Moreover, no tests were applied to confirm post-covid symptoms with greater accuracy. Finally, identification of symptoms among individuals who had not been diagnosed would have enabled a clearer analysis of which manifestations remained after the disease.

## Conclusions

The prevalence of COVID-19 in the sample of farmworkers assessed was similar to the rate observed in the Brazilian population. The most frequently reported symptoms were tiredness, fever, coughing, loss of sense of taste, headache and sore throat. Although none of the participants had been admitted to hospital because of the disease, more than half of these individuals reported post-covid symptoms. Hypertension, cancer, and obesity were associated with COVID-19 diagnosis. These results help to characterize the profile of this disease in a population with reduced access to health services.
